# Comparison of percutaneous cystolithotomy and open cystotomy for removal of urethral and bladder uroliths in dogs: Retrospective study of 81 cases (2014‐2018)

**DOI:** 10.1111/jvim.16577

**Published:** 2022-10-31

**Authors:** Chloé Job, Julie Lecavalier, Marilyn Dunn, Matthieu Gatineau, Jérôme Planté, Jérôme Benamou, Martin Coutellier, Romain Javard

**Affiliations:** ^1^ Surgical Department Centre Vétérinaire DMV Lachine Quebec Canada; ^2^ Internal Medicine Department Centre Vétérinaire DMV Lachine Quebec Canada; ^3^ Département de sciences cliniques, Faculté de médecine vétérinaire Université de Montréal St‐Hyacinthe Quebec Canada; ^4^ Surgical Department Hopital Vétérinaire Centre‐Ville Montréal Montréal Quebec Canada

**Keywords:** cats, cystotomy, dogs, PCCL, stone

## Abstract

**Objective:**

Compare percutaneous cystolithotomy (PCCL) and open cystotomy (OC) for removal of bladder and urethral uroliths.

**Design:**

Retrospective study.

**Animals:**

Client‐owned dogs and cats that underwent PCCL (n = 41) or OC (n = 40) between January 1, 2014 and February 28, 2018 at a referral center.

**Methods:**

Medical records of dogs and cats that underwent a PCCL or an OC were reviewed. History, signalment, physical examination, diagnostic tests, length of the procedure and anesthesia, complications, and duration of hospitalization were recorded.

**Results:**

A total 17 cats (PCCL = 10; OC = 7) and 64 dogs (PCCL = 31; OC = 33) were included. There was no significant difference, regardless of species, in the mean surgical time (45 min [24‐160 min] and 48.5 min [15‐122 min] with *P* = .54 in dogs, *P* = .65 in cats) nor mean duration of anesthesia (90 min [50‐120 min] and 98 min [54‐223 min] with *P* = .87 in dogs, *P* = .08 in cats) in the PCCL and OC groups respectively. Number of uroliths did not affect duration of surgery in either group. Complete urolith removal was achieved in 98% of dogs and cats in both groups. The median hospitalization time was significantly shorter in the PCCL group for dogs (11.3 hours [range 4 to 51.3] in the PCCL vs 56.6 hours [range 7.3 to 96] in the OC group; *P* < .001) but did not differ for cats (24.5 hours [range 8.3 to 30] in the PCCL vs 56.6 hours [range 10.1 to 193.2] in the OC group; *P* = .08).

**Conclusion and Clinical Relevance:**

Bladder urolith removal by PCCL procedure is no longer than OC. Further studies are needed to compare the pain related to procedure between PCCL and OC.

**Abbreviations**
BID: twice a day
LAC: laparoscopic‐assisted cystotomy
NSAID: nonsteroidal antiinflammatory drug
OC: open cystotomy
PCCL: percutaneous cystolithotomy
PO: per‐os
Vs: versus

## 1 INTRODUCTION

Open cystotomy (OC) has traditionally been recommended to remove lower urinary tract uroliths from dogs.^1^, ^2^, ^3^ However, even if OC is considered a routine procedure, it carries risks including clot formation and obstruction of the urethra, intraabdominal adhesions, dehiscence (2%‐3%), incomplete urolith removal (20%), postoperative pain, and postoperative hematuria (50%).^1^, ^4^, ^5^, ^6^ Minimally invasive techniques are recommended for lower urinary tract urolith removal in dogs and cats, voiding urohydropropulsion, intracorporeal lithotripsy, cystoscopic urolith basket retrieval, percutaneous cystolithotomy (PCCL), and laparoscopic‐assisted cystotomy (LAC).^1^, ^4^, ^6^, ^7^, ^8^, ^9^

This PCCL technique was first published in 2011 and is established in the veterinary literature.^1^, ^6^, ^7^, ^10^

There are several advantages of endoscopic assisted techniques compared to OC.^1^ Among these advantages, are better visualization of the lower urinary tract with more complete urolith removal, shorter hospital stays, and more rapid recovery.^1^, ^4^, ^5^, ^10^ Anecdotally, a potential disadvantage to PCCL is the perceived longer procedural and anesthesia times. Despite the growing popularity of minimally invasive procedures in veterinary medicine, there are currently limited studies comparing PCCL to OC at the same institution.

The objective of this study was to compare surgical technique, perioperative and postoperative complications, anesthesia time, surgery time, and length of hospitalization in dogs and cats undergoing PCCL or OC. We hypothesized that length of surgery and anesthesia would be similar for PCCL and OC with similar complication rates however that length of postoperative hospitalization would be significantly shorter in the PCCL group.

## 2 MATERIALS AND METHODS

### 2.1 Case selection

Medical records of all client‐owned cats and dogs that had undergone PCCL or OC for removal of bladder, urethral or both uroliths from the DMV Center between January 6, 2014 and February 28, 2018 were reviewed. Dogs and cats of any age, sex, and size and with any number of bladder or urethral uroliths were included if a PCCL procedure or OC had been performed.

Dogs and cats were included if a complete medical record was available for review, including a 2 week postoperative examination. Signalment, medical history, laboratory (complete blood count, serum biochemistry, and urinalysis) and imaging results and number and type of uroliths were recorded. Length of anesthesia and surgery, number and type of uroliths removed, peri and short‐term postoperative complications (0‐14 days after the procedure) and duration of hospitalization were recorded. Animals were excluded if the medical records were incomplete or if the 2 week postoperative examination was unavailable. All procedures performed without a board‐certified surgeon were excluded.

Criteria used for selecting dogs and cats for PCCL vs OC were owners' preference for a minimally invasive procedure vs open surgery, cost considerations, and availability of the PCCL procedure.

If urethral uroliths were present, retro‐urohydropulsion was performed under general anesthesia using a red rubber catheter (Red Rubber catheter, 3.5; 5; 8 Fr, Covidien, Ilc, Mansfield, Massachusetts, USA). In all cases, uroliths were retropulsed into the bladder preoperatively.

All dogs and cats were anesthetized with propofol (Propoflo, Zoetis Canada Inc, Kirkland, Quebec, Canada −4 mg/kg intravenously IV as needed), midazolam (Midazolam, Pfizer Canada Inc, Kirkland, Quebec, Canada −0.2 mg/kg IV) intubated and maintained on isoflurane. Remifentanil (Remifentanil chlorhydrat, Sandoz Canada Inc. Boucherville, Quebec, Canada) was administrated as a continuous rate infusion (25 μg/kg/hour). The ventral aspect of the abdomen was clipped and aseptically prepared including the prepuce in male dogs.

### 2.2 PCCL procedure

All procedures were performed by the same board‐certified internist (R.J) along with a board‐certified surgeon (M.G, J.B, J.P) and 1 surgical intern. All surgical reports documented rigid normograde cystoscopy (Cystoscope, 2.7 mm, 19 cm, Karl Storz Veterinary Endoscopy, Goleta, California, USA) and flexible urethroscopy (Ureteroscope, Flex‐X2, 3.6 mm, 67 cm, Karl Storz Veterinary, Endoscopy, Goleta, California, USA), allowing complete evaluation of the bladder and urethra. After standard preparation, dogs or cats were placed in dorsal recumbency with the surgical table slightly inclined with the animal's head down, in the Trendelenburg position (15° as described by Trendelenburg). This positioning facilitated identification of the bladder apex. An appropriately sized red rubber urethral catheter was placed in a retrograde manner. Sterile saline was infused until the bladder apex could be palpated and a PCCL procedure was performed as previously described.^10^ Briefly, a 1 cm ventral midline skin incision was performed. Two stay sutures were placed in the right and the left external rectus sheath to retract the body wall with 2‐0 polydioxanone (PDS suture, Ethicon Inc, Somerville, New Jersey, USA). The bladder apex was retracted cranially and a third stay suture was placed at the bladder apex with 3‐0 polydioxanone suture. Two more stay sutures were placed on the right and on the left side of the ventral aspect of the apex of the bladder. A stab incision with #15 or #11 scalpel blade was made into the bladder lumen. A 5‐mm laparoscopic threaded trocar (Smooth trocar‐cannula assembly, 6 mm, Karl Storz Endoscopy, Goleta, California, USA) with a diaphragm was introduced through the stab incision, and directed toward the urethral lumen to maintain a closed system. To remove obvious sediment or small uroliths (<3‐4 mm) prior to cystoscopy, multiple flushes with sterile saline solution (NaCl 0.9% Versol) were performed through the urethral catheter. A rigid 30°‐ and 2.7‐mm cystoscope was advanced and the location and number of uroliths were recorded. Evidence of mucosal lesions including polypoid masses and retained suture material from previous surgeries was identified and biopsied or removed with endoscopic biopsy forceps for biopsies or mosquito forceps for mass removal. A urolith retrieval basket (Gage Nitinol urolith extractor, Modified basket, 2.2 Fr, 115 cm, 8 or 11 mm, Cook Medical, Cook Inc, Bloomington, Indiana, USA) was used to grasp the uroliths and remove them through the trocar. Uroliths larger than the 5‐mm inner diameter of the trocar were entrapped in the urolith basket. The trocar and uroliths were removed from the bladder. Once the procedure was complete, the urethra was inspected with a flexible ureteroscope. As the cystoscope was directed down the urethral lumen, the red rubber catheter was withdrawn and irrigation performed with saline allowing retrograde flush of any remaining urolith fragments. If uroliths were identified, they were retrieved by use of a urolith basket. The ureteroscope was advanced as far down the urethra as possible for male cats and to the proximal aspect of the os penis in small dogs. The bladder incision was closed in 1 or 2 layers at the surgeon's discretion: a simple continuous suture pattern and a Cushing pattern or 2 cruciates sutures with 3‐0 (dogs) or 4‐0 (cats) polydioxanone or poliglecaprone 25 sutures (Monocryl suture, Ethicon Inc, Somerville, New Jersey, USA). The abdominal incision was closed routinely in 3 layers. One non absorbable nylon cruciate suture (Ethilon suture, Ethicon Inc, Somerville, NJ) or surgical glue was used to close the skin incision. A nonadherent dressing (Opsite, Smith&Nephew Medical Ltd, Hull, England) was placed for the duration of the hospital stay.

### 2.3 Cystotomy procedure

The procedure was performed by a board‐certified surgeon (MG, JP, JB) and surgical interns. Dog or cat was placed in dorsal recumbency after a standard aseptic preparation of the abdomen. Sterile saline was infused through the red rubber catheter placed as described in the PCCL procedure section. The OC was routinely performed as previously described.^2^ The skin incision length varied from 2 to 7 cm depending on the size of the dog or the cat. The bladder and abdominal incisions were closed as described for the PCCL procedure. An adhesive dressing was placed for the duration of the hospital stay.

### 2.4 Complications

Intraoperative complications were defined as complications noted during the surgical procedure. They were classified as severe or minor. Severe complications were defined as complications which could be life‐threatening or which needed surgical conversion from PCCL to OC. Minor complications were associated with an increased surgical time that did not require surgical conversion from PCCL to OC.

Complications were considered short‐term if they occurred within 3 days and medium‐term between 3 days and 2 weeks postoperatively. Postoperative complications were classified as major or minor according to the definitions and criteria proposed by Cook et al for orthopedic studies.^11^ Minor complications required no treatment while major complications required surgical or medical therapy for resolution (wound dehiscence, remaining uroliths, wound infection, urethral obstruction) or complications which could be life‐threatening.

Uroliths were submitted for analysis to the Idexx Laboratory (Idexx Laboratories Canada Corp. 1345 Denison Street, Markham, Ontario, Canada) or Minnesota Urolith Center (Minnesota Urolith Center, University of Minnesota, Saint Paul, Minnesota, USA). One crushed urolith was submitted for aerobic bacterial culture and sensitivity testing.

### 2.5 Postoperative management

All dogs and cats with radio‐opaque uroliths had a lateral and ventro‐dorsal abdominal radiographs taken immediately after surgery to confirm urolith removal defined as no uroliths in the bladder nor urethra directly visualized during the PCCL procedure and on postoperative abdominal radiographs (when applicable). For the OC procedure, complete urolith removal was characterized by absence of uroliths on postoperative abdominal radiographs (when applicable). If uroliths were seen within the urinary tract, a revision surgery was performed. For animals with radiolucent uroliths, postoperative radiographs were not taken. Meloxicam (Metacam, injectable susp for dogs, Boehringer Ingelheim, Pierrefonds, Quebec, Canada −0.2 mg/kg in dogs and 0.3 mg/kg in cats) SC was administered after the procedure then prescribed (Metacam, oral suspension for dogs, Boehringer Ingelheim, Pierrefonds, Quebec, Canada −0.1 mg/kg PO in dogs and 0.15 mg/kg PO in cats) for 0 to 7 days in the PCCL group (administrated as needed) or 7 to 10 days in the OC group (consistently given). Analgesic drugs used included sublingual buprenorphine (Vetergesic, Ceva Animal Health Inc, Cambridge, Ontario, Canada −15 μg/kg q12h for 3‐5 days) or long‐acting buprenorphine (Simbadol, Zoetis Canada Inc, Kirkland, Quebec, Canada −0.12 mg/kg SQ once) in cats and tramadol (3‐5 mg/kg q12h for 3‐5 days PO) or gabapentin (Neurontin, Pfizer Inc, Kirkland, Quebec, Canada −10 mg/kg q12h for 3‐5 days) in dogs. Dogs and cats were discharged with prophylactic antimicrobials at the clinician's discretion and adjusted according to results of urolith and urine cultures.

### 2.6 Short‐term follow‐up

Dogs and cats were initially reevaluated by 1 of the authors or the referring veterinarian at the time of suture removal, 12 to 14 days postoperatively. The surgical site was evaluated and a history and physical exam were performed. Diet and urolith prevention protocols were discussed and instaured based on urolith analysis.

### 2.7 Statistical analysis

Numeric values are summarized as mean ± SD. Unpaired Student's *t*‐test was used to compare the length of anesthesia, length of surgery, the age and weight between the 2 groups (OC and PCCL). Homogeneity of variance was tested with an *F* test. Welch's unpaired *t*‐test was used to compare the hospitalization times between the 2 groups because of the disparity in variances (heteroscedasticity). One‐way ANOVA with Welch's correction was used to determine whether there were any statistically significant differences between the hospitalization time, lengths of surgery and anesthesia for PCCL and OC in function of the number of uroliths. Data failed Kolmogorov‐Smirnov normality test and passed Brown‐Forsythe test for homogeneity of variances. Welch's modification was used because of the sample size disparity. When necessary, posthoc was done using Dunnet's T3 multiple comparisons test. Fisher's exact test was used to determine if there was a nonrandom association between the type of surgery and complication rate. A 2‐tailed test was chosen given the potential nondirectional statistical difference in the proportions. Values of *P* < .05 were considered significant for all comparisons. Statistical analysis was performed using a software adapted for nonlinear regression, and scientific graphing (GraphPad Prism version 8.3.1 for Mac Software, San Diego, California, USA, www.graphpad.comfor).

## 3 RESULTS

Between January 1, 2014 and February 28, 2018, 81 dogs and cats (17 cats and 64 dogs) met the inclusion criteria and underwent surgery for bladder urolith removal at the DMV Center.

### 3.1 Animals

OC was performed in 40 dogs and cats and 41 underwent PCCL. In the PCCL group, there were 10 cats (9 domestic short hair [DSH] and 1 Maine Coon, 7 neutered males, and 3 spayed females) and 31 dogs (24 neutered males, 3 spayed females, and 4 intact males). In the OC group, 7 cats (1 Maine coon, 1 Rex Devon, 1 Persian, 4 DSH with 4 males neutered, and 3 males intact) and 33 dogs (19 males neutered, 7 females spayed, 6 males intact, and 2 females intact) were included. The median age and weight in the PCCL group was 8 years for dogs (range, 0.7‐13.5 years) and 8.8 years for cats (range, 1.6‐13 years) and 7.9 kg for dogs (range, 3.4‐74.4 kg) and 5.65 kg for cats (range, 3.4‐8.6 kg). The median age and weight for dogs and cats in the OC group was 6.9 and 4.6 years respectively (range, 1.6‐13.8 and 2.5‐14.5 years respectively) and 12.7 and 6.36 kg respectively (range, 2.5‐40.7 kg for dogs and 4.9‐8.9 kg for cats). There was no statistical significant difference in age (*P* = .16) and weight (*P* = .27) between both groups for both species. Dog breeds in the PCCL group included Yorkshire terrier (n = 6), Miniature Schnauzer (n = 5), mixed (n = 3), Pug (n = 3), Havanese (n = 3), Shih Tzu (n = 3), Pomeranian (n = 2), Bullmastiff (n = 1), Lhasa Apso (n = 1), Shetland Shepherd dog (n = 1), Siberian Husky (n = 1), Doberman Pinscher (n = 1), and English Bulldog (n = 1). Dog breeds in OC group included mixed breed (n = 6), Miniature Schnauzer (n = 4), Pug (n = 3), Yorkshire terrier (n = 2), Shih Tzu (n = 2), Chihuahua (n = 2), English Bulldog (n = 2), Labrador retriever (n = 2), Havanese (n = 1), English Springer (n = 1), Lhasa Apso (n = 1), Eurasier (n = 1), Siberian Husky (n = 1), Pitt Bull (n = 1), Basenji (n = 1), Miniature Poodle (n = 1), Labernese (n = 1), and Standard Poodle (n = 1).

### 3.2 Preoperative data

Abdominal radiographs were performed in 90.0% of dogs and cats (36/40 6 cats and 30 dogs) in the OC and 73.2% (30/41 7 cats and 23 dogs) in the PCCL group. A urinary tract ultrasound was done in 18% (7/40 2 cats and 5 dogs) in the OC and 46.3% (19/41 4 cats and 15 dogs) in the PCCL group. A complete blood count and serum biochemistry panel were available in 85.0% (34/40 6 cats and 28 dogs) in the OC and 97.5% (40/41 9 cats and 30 dogs) in the PCCL group. A urinalysis was performed in 62.5% (25/40 6 cats and 19 dogs) in the OC and 90.2% (37/41 9 cats and 18 dogs) in the PCCL group. A urine culture and sensitivity obtained by cystocenthesis was available in 70.7% (29/41 6 cats and 23 dogs) in the PCCL and 42.5% (17/40 4 cats and 13 dogs) in the OC group. Preoperatively, no data was available for 4 dogs and 1 cat in the OC group and 1 dog and 1 cat in the PCCL group. These dogs or cats were excluded of the statistical analysis. In the OC group, 65.7% (23/35) and 67.5% (27/40) dogs or cats in the PCCL group did not have azotemia and had serum potassiums within reference range. In the OC group, 8/35 (23% 2 cats 6 dogs) had increased creatinine and urea, 2/35 dogs had an increased urea alone, 1/35 dog an increased creatinine alone and 1/35 cat was hyperkalemic. In the PCCL group, 6/40 (15% 4 cats and 2 dogs) were azotemic, 4/40 (1 cat and 3 dogs) had an increased urea and 2/40 cats were hyperkaliemic and 1 dog was hypokaliemic. No significant difference in renal biomarkers was found between the PCCL and OC groups (*P* = .49).

### 3.3 Intraoperative data

#### 3.3.1 Surgical procedures

For the PCCL group, all the procedures were performed by the same internist, in combination with the same surgeon for 93% of the cases. Evaluation of the entire urethra was possible in all females (n = 4 dogs and n = 3 cats) and all male dogs ≥6 kg (13.2 lbs) (n = 22 dogs). For male cats, the first two third of the urethra was evaluated and for dogs <6 kg, the urethra was inspected until the penile bone. The median anesthesia time was 90 min (range, 54‐223 min) in the PCCL group and 98 min (range, 50‐210 min) in the OC group (*P* = .93). The median surgical time was 48.5 min in OC (range, 15‐122 min) and 45 min in PCCL group (range, 24‐160 min) (*P* = .76). There was no significant difference in length of anesthesia nor length of surgery between the OC and PCCL groups regarding each species (dogs: 98 min in PCCL group [range, 60‐1020 min] and 101 min in OC group [range, 54‐223 min] *P* = .87; cats: 100 min in PCCL group [range, 50‐165 min] and 102.5 min in OC group [range, 70‐159 min] *P* = .08 in anesthetic time and dogs: 49.5 min in PCCL group [range, 15‐122 min] and 45 min in OC group [range, 24‐160 min] *P* = .54; cats: 50.5 min in PCCL group [range, 29‐70 min] and 45 min in OC group [range, 30‐84 min] *P* = .65 in surgical time). The surgical time was not significantly different in dogs or cats with < or ≥10 uroliths (*P* = .37) in the PCCL and OC groups (Table 1).

**TABLE 1:** Summary of anesthetic, surgery and hospitalization times for dogs and cats for the PCCL and OC groups

	Weight (kg) (median)	Age (years) (median)	Anesthetic time (min) (median)	Surgery time (min) (median)	Hospitalization time (hours) (median)
PCCL Cat = 10	5.65 (3.4‐8.6)	8.8 (1.6‐13)	100 (50‐165)	45 (30‐84)	24.5 (8.3‐30)
OC cats = 7	6.36 (4.9‐8.9)	4.6 (2.5‐14.5)	102.5 (70‐159)	50.5 (29‐70)	56.6 (10‐193.2)
PCCL dogs = 31	7.9 (3.4‐74.4)	8 (0.7‐13.5)	90 (60‐1020)	45 (24‐160)	11.1 (4‐51.3)
OC dogs = 33	12.7 (2.5‐40.7)	6.9 (1.6‐13.8)	101 (54‐223)	49.5 (15‐122)	28.2 (7.3‐96)
Significant difference	No (dogs *P* = .53; cats *P* = .35)	No (dogs *P* = .23; cats *P* = .15)	No (dogs: *P* = .87; cats: *P* = .08)	No (dogs: *P* = .54; cats: *P* = .65)	Yes for dogs only (*P* < .0001), (cats: *P* = .08)

In total, 63 dogs and 16 cats were discharged from the hospital (all except 1 dog in the PCCL group and all except 1 cat in the OC group). In the PCCL group, 10 cats and 18/40 dogs (70.0%) were discharged the same day of the procedure, whereas only 1 cat and 1 dog (2/39) were discharged the day of the procedure in the OC group. A significant difference was noted in the hospitalization time between the 2 groups: postoperative time was found to be 18.1 ± 3.8 hours shorter for the PCCL compared to OC group (*P* < .01). In dogs, the median hospitalization time in the PCCL group was 11.1 hours (range, 4‐51.3 hours) and 28.2 hours (range, 7.3‐96 hours) in the OC group. In cats, the median hospitalization time was 24.5 hours (range, 8.3‐30 hours) and 56.6 hours (range, 10‐193.2 hours) in the PCCL and the OC groups respectively. Median hospitalization time was significantly different between the OC and PCCL groups (*P* < .01) in dogs but not in cats (*P* = .08). During the PCCL procedure, 2 dogs had biopsies (1 transitional cell carcinoma of the bladder and 1 mass not analyzed due to owner preference). No biopsies were performed during the OC procedures.

#### 3.3.2 Complications

There were no major intraoperative complications in the PCCL group, with no need for conversion to an OC. Postoperative abdominal radiographs were performed in both groups for all dogs and cats with radio opaque uroliths, which was the vast majority of animals (24 dogs and 8 cats in the PCCL group and 32 dogs and 5 cats in the OC group). In other dogs and cats, no radiographs nor abdominal ultrasound were performed postoperatively because of gas interference directly after the procedure. No dog and no cat had uroliths visible on postoperative radiographs in PCCL group. The only dog with incomplete urolith removal in the PCCL group (1/40) was an intact male (Bullmastiff of 74.4 kg) with a large number of cystine uroliths (>100). In this particularly long procedure (160 min), a few small uroliths fragments (<1 millimeters of diameter) were left in the bladder. The dog urinated well after the PCCL and remained asymptomatic with no urolith fragments visible on follow‐up ultrasounds during the 2 year follow‐up period.

During the short‐term follow‐up in the PCCL group, 1 dog died from refractory epileptic seizures, no other major complications occurred. At the 2 week examination, minor postoperative complications were reported by owners in 32% (13/41) of animals in the PCCL group including lower urinary tract signs during the first 72 hours (pollakiuria 5/17, stranguria 5/17, periuria 2/17, hematuria 1/17) and wound inflammation (redness during the first 5 days after surgery) in 3/17 animals.

During the OC procedure, 1 cat with severe azotemia and electrolyte disturbances (creatinine 501 mmoL/L [range, 71‐212 mmoL/L] and urea >46.4 mmoL/L [range, 5.7‐12.9 mmoL/L]) died from cardiac arrest (2.5%) during the procedure despite preoperative stabilization (fluid therapy and analgesia) and urethral catheterization. In this group, 1 dog presented a minor complication with an abdominal urolith noted during the post operative radiographs. One dog and 1 cat presented major short‐term complications: the dog had uroabdomen 24 hours after surgery (partial necrosis of the bladder) and the cat had a urethral obstruction 48 hours after surgery due to a persistent urethral urolith missed on direct postoperative radiographs. The dog with uroabdomen was initially presented for hematuria without urethral obstruction. The uroabdomen might have been caused by a traumatic catheterization. The cat was initially presented with urethral obstruction and treated by urethral catheterization (preoperative radiographs confirmed that all uroliths were in the bladder). Both underwent a successful surgical revision. The overall intraoperative success rate for complete urolith removal was 97.2% in the PCCL (40/41) and 97.2% (36/37) in the OC group.

At the 2 week examination, minor postoperative complications were reported by owners in 45% (18/40) in the OC group. Minor complications recorded consisted of lower urinary tract signs (hematuria 8/24, pollakiuria 5/24, stranguria 4/24 and periuria/incontinence 2/24), wound inflammation in 5/24 animals (for the first 8 days postoperatively) and general discomfort in 6 dogs or cats (for the first 10 days postoperatively). In both groups, all minor complications resolved within 10 days after the procedure.

No significant difference was found between the 2 groups regarding intraoperative complications (*P* = .36), and postoperative minor and major complication rates (*P* = .36).

## 4 DISCUSSION

This report compares PCCL and OC in the same institution. PCCL was successfully performed with few complications and no difference in anesthetic and surgical times regardless of dog or cat size, sex and number of uroliths and none underwent conversion to OC.

The median surgical time for PCCL in our study was 45 min which was shorter than previously described (66 min in a study of 27 dogs and cats and 96 min in a recent study).^1^, ^10^ Studies comparing LAC to OC have reported significantly longer procedural times for LAC.^12^, ^13^, ^14^, ^15^ These differences could be due to the type of practice (academic vs private practice) and the time between studies allowing a certain learning curve. Surprisingly, the number of uroliths did not affect surgical times in our study. However, for extreme cases, such as the longest procedure in our study, a surgical time of 160 min was reported in a dog with >100 cystine bladder uroliths. Our study showed that PCCL can be safely and efficiently performed in dogs and cats of varied sex, weight, and with any urolith burden. The authors believe that the Trendelenburg position^16^ allowed uroliths to fall near the bladder apex facilitating their removal and the abundant flushing at the beginning of the procedure accelerated urolith removal and might explain the shorter procedural times as compared to other studies.^1^, ^10^ We also believe that the combination of a board‐certified surgeon and internist with knowledge and experience of the procedure helped achieve shorter surgical and anesthesia times.

In our study, complete urolith removal was achieved in 97.6% (40/41) of dogs (40) and cats (10) in the PCCL group. This is similar to a previous study, in which there was 1/27 dogs (3.7%) with incomplete urolith removal by PCCL.^1^, ^7^ In LAC, incomplete urolith removal was described in 3/50 dogs (6%).^12^ In our study, there was 1 dog with cystine urolith fragments left intentionally in the bladder following PCCL, as their removal would have resulted in a prolonged procedural time. Given the small size of the fragments (<1 mm) it was expected that they would be evacuated from the bladder/dissolve following resolution of androgen dependent cystine crystalluria. No urolith recurrence was documented throughout the 2 year follow‐up. The rate of persistence of small urolith fragments in the OC group remains uncertain as they are unlikely to be seen nor palpated during surgery.

In the OC group, 1 cat presented an obstruction 48 hours postoperatively due to a urethral urolith missed on postoperative radiographs. Our results compare favorably to other studies that have reported urolith persistence rates after OC of 14% to 20% in dogs and up to 20% in cats.^2^, ^5^ The true persistence rate could have been underestimated in the OC group because of the lack of cystoscopic evaluation of the urinary tract at the end of the procedure, especially for small fragments typically not visible on radiographs. Though this could falsely decrease the percentage of incomplete uroliths removed in the OC group, these small fragments are unlikely to have clinical significance.

One dog had a free abdominal urolith after the OC procedure. This minor complication was avoided in the PCCL group likely because of the pexy of the bladder to the abdominal wall limiting peritoneal cavity leakage during the procedure.

PCCL allows direct visualization of the lower urinary tract and image‐guided urolith removal. Saline distension during PCCL combined with the magnification of the camera, allows better inspection of the bladder and urethral lumen. In particular, bladder biopsies can be easily performed with little added surgical time.^1^, ^2^, ^5^, ^7^

In this study, the surgical team did not consider it necessary to convert any PCCL procedure into OC.^1^, ^10^ This is in contrast with reported intraoperative complications during LAC procedure which occurred in 19.2% of cases leading to OC in 3 dogs.^2^, ^17^ Conversion to OC during LAC is required in 5/50 (10%) dogs.^12^ However, this could be due to dog or cat and surgeon related factors that could vary between institutions. PCCL might provide other benefits as well when compared to LAC. In LAC, the abdomen must be insufflated to allow visualization of the bladder. Abdominal insufflation might be painful for the dog or cat and further studies are warranted to better assess this aspect.^18^ The median surgical time of LAC is much longer than the PCCL procedure time in the present study (respectively 80 vs 45 min), representing another potential advantage of PCCL over LAC.^12^ However, further studies are required to support this. Hospitalization time is similar for LAC in comparison with OC whereas in the present study, dogs and cats in the PCCL group had a shorter hospitalization time compared to OC.^12^, ^13^ However, other variables between studies and institutions as well as attending clinician perception might also have contributed to this apparent difference and should be investigated.

In this study, except for emergencies, most of the dogs and cats in the PCCL group were admitted as an out‐patient procedure. Dogs and cats in the PCCL group were admitted the morning of surgery and discharged the same day, which was greatly appreciated by owners. In contrast, in the OC group, only 1 dog and 1 cat were discharged the same day as the procedure and 33% of dogs and cats were hospitalized for >24 hours after surgery. Further studies are needed to compare hospitalization times for equivalent populations (stable vs unstable dogs or cats). However, it is interesting to note that no significant difference was found in increased renal blood markers between the OC and PCCL groups (34% and 32% respectively). Evaluation of hospitalization time is an important factor not only because of its effect on cost, but also because of the potential effect on dog or cat morbidity. This has been extensively investigated in human laparoscopic surgery, which reduces cost as well as iatrogenic complications and dog or cat morbidity.^18^, ^19^ Although this has not been well evaluated in veterinary surgery, it is possible that a similar relationship exists in companion animals. Finally, a shorter hospitalization time might decrease dog or cat and client stress and anxiety that arises from being separated.

Another advantage of PCCL is likely decreased postoperative pain compared with a standard OC. The small cutaneous and muscular incision and the limited bladder manipulation is believed to reduce inflammation and peri and postoperative pain.^1^, ^7^, ^18^ However, postoperative comfort depending on the technique used was not properly evaluated in this study and further investigation with standardized pain scales are needed.

This study has some limitations related its retrospective nature. All the PCCLs were performed by the same internist in combination with the same surgeon for 93% of cases, limiting the bias associated with the change in operators. In the OC group, the 3 surgeons performed an equal number of OC, however surgical interns were involved in some of these surgeries which was not always specified in the medical files. Despite being under the direct supervision of a board‐certified surgeon, the surgical and anesthetic times might have been increased in the OC group. Small sample size, especially in cats, might have led to decreased statistical power, which could explain the lack of a significant difference in hospitalization times between the 2 groups for cats (type II error). Hospitalization time has to be carefully interpreted as the perception that PCCL is a day procedure and OC requires hospitalization might have influenced hospitalization times. Moreover, based on the scheduling for these procedures, comparison of hospitalization times is likely biased by the design of this study and no objective assessments of postoperative pain nor other parameters such as blood markers was assessed for determining time of discharge. Pain was not evaluated in this study because of a lack of a standardized pain scale. Moreover, the postoperative analgesia was not standardized between groups, preventing any sort of comparison of postoperative pain between groups. A prospective comparison of postoperative pain between the 2 procedures using objective criteria is warranted. The absence of long‐term follow‐up for both groups and evaluation of urolith recurrence are lacking.^20^, ^21^, ^22^, ^23^ Moreover, the absence of conversion of PCCL to OC has to be interpreted with caution due to the fact that different thresholds for conversion might vary across institutions and studies, making it difficult to compare conversion rates.

Finally, the cost of equipment for the PCCL procedure and the time required to prepare and clean the operating room might be a limiting factor for some institutions.

In our study, length of surgery and anesthesia were not significantly different for PCCL and OC and hospital stays were significantly shorter in the PCCL group for dogs. We believe that PCCL can be safely and efficiently performed in dogs and cats regardless of weight, sex and with any number of uroliths. Complete urolith removal was achieved in 97.2% of dogs and cats by PCCL. Prospective randomized clinical trials to compare postoperative comfort and urolith recurrence rates between PCCL and OC are needed to better elucidate other potential benefits of PCCL over OC.

## CONFLICT OF INTEREST DECLARATION

Authors declare no conflict of interest.

## OFF‐LABEL ANTIMICROBIAL DECLARATION

Authors declare no off‐label use of antimicrobials.

## INSTITUTIONAL ANIMAL CARE AND USE COMMITTEE (IACUC) OR OTHER APPROVAL DECLARATION

Authors declare no IACUC or other approval was needed.

## HUMAN ETHICS APPROVAL DECLARATION

Authors declare human ethics approval was not needed for this study.
